# Clinical research and medical care: towards effective and complete integration

**DOI:** 10.1186/1471-2288-15-4

**Published:** 2015-01-09

**Authors:** José A Sacristán

**Affiliations:** Clinical Research Department, Lilly Spain, Madrid, Spain

**Keywords:** Research, Medical care, Patient, Patient-centered care, Preferences, Patient-reported outcomes, Randomized clinical trials, Observational studies, Evidence-based medicine, Bioethics

## Abstract

**Background:**

Despite their close relationship, clinical research and medical care have become separated by clear boundaries. The purpose of clinical research is to generate generalizable knowledge useful for future patients, whereas medical care aims to promote the well-being of individual patients. The evolution towards patient-centered medicine and patient-oriented research, and the gradual standardization of medicine are contributing to closer ties between clinical research and medical practice. But the integration of both activities requires addressing important ethical and methodological challenges.

**Discussion:**

From an ethical perspective, clinical research should evolve from a position of paternalistic beneficence to a situation in which the principle of non-maleficence and patient autonomy predominate. The progressive adoption of “*patient-oriented informed consent*”, *“patient equipoise*”, and “*altruism-based research*”, and the application of *risk-based ethical oversight*, in which the level of regulatory scrutiny is adapted to the potential risk for patients, are crucial steps to achieve the integration between research and care.

From a methodological standpoint, careful and systematic observations should have greater relevance in clinical research, and experiments should be embedded into usual clinical practice. Clinical research should focus on individuals through the development of patient-oriented research. In a complementary way, the integration of experiments into medical practice through the systematic application of “point of care research” could help to generate knowledge for the individuals and for the populations.

**Summary:**

The integration of clinical research and medical care will require researchers, clinicians, health care managers, and patients to reevaluate the way they understand both activities. The development of an integrated learning health care system will contribute to generating and applying clinically relevant medical knowledge, producing benefits for present and future patients.

## Background

Clinical research and medical care are closely related activities and represent two sides of the same coin. Acts of patient care are analogous to experiments as each patient begins in a baseline state, receives an intervention, and has an outcome [1]. Furthermore, the ultimate goal of research is to generate knowledge capable of improving the patient’s health outcomes in medical practice. Despite their close relationship, clinical research and medical care have grown apart and nowadays are considered independent activities, separated by clear boundaries.

There are ethical, methodological and organizational barriers that explain this separation. The purpose of clinical research is to produce generalizable knowledge useful for future patients, while medical care aims to promote the well-being of individual patients [2]. This dichotomy creates an ethical conflict for physicians playing dual role as doctors and as researchers. As doctors, they must keep the patient’s interest foremost, but as researchers, they must subordinate the individual patient’s interests to the general interests of the community [3–7]. In addition, clinical research objectives and methods are oriented towards identifying the best intervention for the “average” patient and rarely have a patient-centric focus. Finally, clinical research has become a complex and sophisticated process, involving much dedication of researchers who are increasingly specialized. In health care systems where the main priority is the provision of care, it may be difficult for doctors to dedicate time to research [8].

The ongoing changes within health care systems may contribute to the progressive convergence between research and care. Clinical research is moving its focus towards the assessment of the effectiveness of health interventions under the conditions of routine clinical practice [9]. Some examples of this evolution are the development of Comparative Effectiveness Research [10] and Real World Evidence movements, the creation of the Patient Centered Outcomes Research Institute (PCORI) [11], and the increasing interest in measuring patient-reported outcomes (PRO) such as functional status, quality of life and patient satisfaction [12].

Medical care is also incorporating typical elements from clinical research. For example, the implementation of clinical practice guidelines has encouraged the establishment of standards and protocols in medicine; the increasing number of litigations has led to more defensive medicine practice [13], where obtaining patient’s informed consent has become an usual procedure; and the development of information technologies such as electronic health records allows the systematical analysis of data from thousands of patients and facilitates the decision-making process [14].

Although all these changes are helping to enhance the relationship between clinical research and medical care, there are important ethical and methodological barriers that prevent their full integration. This work analyzes these challenges and proposes some solutions to overcome them.

## Discussion

### Ethical barriers and potential solutions: from paternalistic beneficence to autonomy and non-maleficence

Thirty five years ago, the Belmont Report established a formal distinction between clinical research and medical care [15] and emphasized the different purpose of both activities. In order to overcome the ethical conflict faced by the physicians in their dual role as doctors and researchers, three “solutions” have been proposed: 1) the obligation of informed consent [16]; 2) the requirement of “clinical equipoise” [17]; and 3) the therapeutic orientation of randomized controlled trials [18]. It is noteworthy that the way to interpret and to apply these solutions reflects a paradigm based on the ethical principles of beneficence, paternalism and physician’s authority; a model that does not take into account the evolution towards a *Patient Centered Medicine* where the patient’s goals, preferences and values play a crucial role [19]. The leap from the current model to a new model based on the principles of non-maleficence and autonomy may help to successfully integrate research and care. But this leap requires the reassessment of the “three solutions” (Table 1):

**Table 1:** **Reassessment of the “three solutions” and associated ethical principles in the old and new models of clinical research and medical care**

“Traditional Model”: separation between clinical research and medical care	“New model”: integration of clinical research and medical care
• Researcher-oriented informed consent and high level of oversight (paternalistic beneficence)	• Patient-oriented informed consent (autonomy) and risk-based ethical oversight (no maleficence)
• Clinical equipoise (physician authority)	• Clinical equipoise + Patient equipoise (autonomy)
• Therapeutic orientation (beneficence)	• Altruism-based research (autonomy)

1. From researcher-oriented informed consent to patient-oriented informed consent:
  The purpose of the *Informed Consent Document* is to ensure that individual patients can control whether or not they participate in clinical research, and that they only participate if the research is consistent with their values, interests, and preferences [16]. One of the main factors that obstruct the integration of clinical research and medical care is the current regulation systems that require excessively detailed inform consent documents, even for trials comparing widely accepted treatments [20]. In practice, the information contained in informed consent documents is extensive, complex, and difficult to understand [21]. Many patients agree to participate in randomized controlled trials because of their trust in their physicians. Some authors have indicated that “*although the Informed Consent Document was a major stride towards protecting the rights and dignity of patients, the process may in some instances be perverted into protecting the physician rather than the patients*” [22].
  The development of the *Patient Centered Medicine* movement can facilitate the transition from the principle of beneficence to the principles of non-maleficence and autonomy. Within this context, non-maleficence represents the adaptation of regulatory research requirements to the risks for patients. The degree of monitoring and the extent of oversight should be adapted to the level of risk of the studies [23, 24]. The adoption of *risk-based ethical oversight* implies that for specific comparative effectiveness research studies may be justifiable to proceed with a streamlined informed consent process and that under specific circumstances the consent may be eliminated [25, 26].
  The question is what kind of trials are those involving “low risk” conditions? Considering the established classification into exploratory and pragmatic studies [27], it can be argued that, in general, suitable conditions to integrate clinical research and medical care are present in many pragmatic trials comparing standard-of-care interventions [28]. Conversely, “low risk conditions” may not be applicable to exploratory trials, which are aimed at increasing our understanding the potential therapeutic effects of a new drug. These trials usually include a placebo arm, and are conducted according to a strict predefined protocol. The “investigational” component prevails over the “medical care” objective, and patients are considered as “investigational subjects”. Selected patients, comparators, time-frame, and outcomes, among other factors, are oriented toward regulatory approval and do not always reflect the routine clinical practice environment. Consequently, the integration between clinical research and medical care may not be feasible in such trials.
2. From clinical equipoise to clinical and patient equipoise:
  *Clinical equipoise* exists when “there is no consensus within the expert clinical community about the comparative merits of the alternatives to be tested” [17]. It is often difficult to assess whether clinical equipoise actually exists, who the experts are, or on what end points is equipoise being assessed [29]. A situation of clinical equipoise is especially difficult to achieve in placebo-controlled clinical trials, and also when a new drug under development is compared with a commercialized drug. In addition, it is necessary to reflect on the meaning of clinical equipoise within the context of personalized research, when growing evidence is showing the heterogeneity of the responses of different type of patients to the same treatment [30], and in a health care system where patients’ preferences should play an key role [31].
  A classical article describing the impact of patient preferences in clinical research stated that *“…although the treatments being compared were not known to differ in terms of their efficacy, they did differ in terms of their impact on the patients’ lives, and physicians knew this. It is not enough for the physician to have no reason to prefer one treatment over the other; in addition, there must be no reason for the patient to prefer one treatment*” [4]. Therefore, to include a patient in a randomized controlled trial, in addition to the principles of clinical equipoise (from the scientific community) and physician equipoise, it should be confirmed that the patient does not prefer any of the treatments being evaluated (i.e., there should be “*patient equipoise”*). This will require a shift from physician’s authority to patient’s autonomy. The increasing adoption of electronic health records and electronic patient decision aids could facilitate patient-physician communication and shared decisions [32, 33] by integrating patients’ preferences not only into medical decision making but also into clinical research. Ideally, electronic patient decision aids should help doctors to assess whether *patient equipoise* exists.
3. From therapeutic orientation to altruism:
  The “*therapeutic orientation*” of randomized controlled trials substantiates the tendency to justify patients’ participation in randomized controlled trials based on the therapeutic benefits that they may derive from participating in such studies. The therapeutic orientation gives way to “*therapeutic misconception*”, defined as the tendency for patients/subjects to confuse their participation in clinical trials with personalized medical care [34, 35]. Usually, the information included in Informed Consent Document overstates the potential direct benefits for the patients [36].
  Although participation in a new randomized controlled trials is sometimes the only way for a patient to receive a new investigational drug (e.g., for diseases for which there are no alternatives, or for which current alternatives are not efficacious), it is not possible to guarantee that participation in a randomized controlled trial results in better health outcomes for the patients. Reviews of this subject have not found consistent evidence for the existence of a beneficial trial effect [37, 38]. A research model based on the principles of autonomy and non-maleficence requires that patients receive complete and understandable information about the study; and that they understand that altruism and moral obligation, and not potential clinical benefits, should guide their participation in clinical research [39, 40]. The acceptation by society that patients have a “moral duty” to participate in research is a critical step to create an integrated learning health care system, where boundaries between clinical research and medical care will progressively disappear.

### Methodological challenges and potential solutions: the methods in translation

From a methodological point of view, the different objectives of clinical research and medical care represent an obvious barrier to integrate both activities. Evidence-Based Medicine (EBM) and Patient-Centered Medicine are two movements that have emerged with great force in health systems in recent years. EBM has its conceptual anchor in research, while Patient Centered Medicine has it in medical care [41]. EBM focuses on the generalization of the results, the aggregation of data, the analyses of commonalities, and the evaluation of the efficacy in average patients. Randomized controlled trials have become the cornerstone for EBM.

PCM proposes to look back to the individual patient, understood as a person. It “*tries to provide the best health care to every patient, under the conditions of clinical practice, taking into account their objectives, preferences and values as well as available economic resources”* [42] *.* The development of Patient Centered Medicine requires the growth of a patient-oriented research focused on the “individualization” of results, the disaggregation of data, the analysis of the differences in subgroups and individuals, the study of heterogeneity, the analyses of exceptions or anomalies, the identification of the best option for each patient [43], and the application of a hypothetic-deductive logic where trials are considered exploratory and observations confirmatory [44, 45]. If the randomized controlled trial is the basis for EBM, careful individual observations are the foundation for patient-oriented research [41].

**Figure 1:**
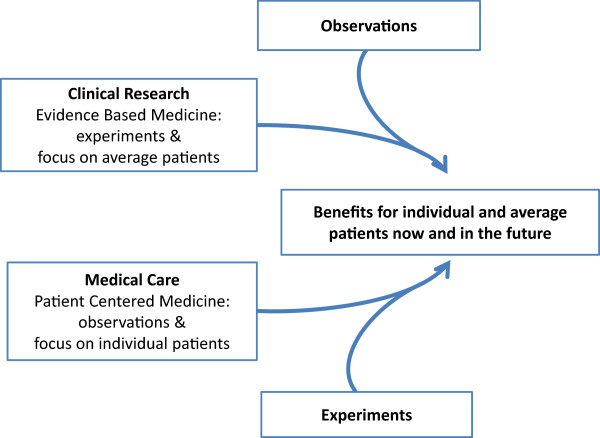
**Methodological proposals to integrate clinical research and medical care.** Abbreviations: EBM = Evidence Based Medicine; PCM = Patient Centered Medicine.

The integration of clinical research and medical care demands that EBM and Patient Centered Medicine change their traditional reference points and find common ground. Both EBM and Patient Centered Medicine can help generate knowledge and improve health outcomes for individual and average patients, today and in the future [41]. In other words, observations should have greater relevance in the clinical research process, and experiments should be integrated into usual medical practice (Figure 1):

1. Integration of observations into clinical research

Progress towards patient-oriented research must have a clear focus on individual patients and be sustained by the development of a research methodology that rediscovers the crucial role of *careful individual observations*. Although the observations of individual cases occupy the lowest place in the hierarchic classification of evidence, they provide first line evidence [46, 47]. The exceptions and unexpected findings often direct research into new fields and sometimes the “evidence” that comes from individual cases and “anecdotes” may be deemed “confirmatory” and can change clinical practice in an instant [48, 49]. The increasing use of electronic health records may help to identify these “exceptions” and anomalies (e.g., unexpected benefits or adverse effects), to analyze the factors associated with their appearance, and alert doctors on the need to specifically monitor such exceptions. Electronic health records may also help to assess how and in which types of patients’ interventions are applied in clinical practice, to identify subgroups of patients, and to classify patients according to risk factors and comorbidities [45].

Khun’s description on the discovery of the first antidepressant represents an excellent example of how careful observation of patients has been central to the process of clinical research: “*I’ve never used ‘double-blind, controlled studies’ versus placebo, standardized rating scales, or statistical treatment of data from a large number of patients. Instead, I examined each patient individually every day, often several times, and asked them questions over and over. Many of my patients were also under the observation of my assistants and nurses, and I always took their suggestions and criticisms very seriously* [50]”. In addition, careful observations have demonstrated a high value in the context of “experimental research”. As noted by Dr Crofton, a Member of the Medical Research Council who participated in the first clinical trial [51], “*…Randomized trials were not intellectually stimulating. Our greatest intellectual challenge with tuberculosis research was to identify the reasons why the treatment failed. The results of randomized trials, together with detailed investigation of drug resistance in individual patients and the appropriate organization of services, allowed our team to reach one hundred percent recovery from pulmonary tuberculosis, the most common form of the disease and one that not long ago killed half of the patients who suffered from it*”.

In an attempt to systematically analyze case reports, some authors have proposed to conduct “formal case studies” [52] in which certain subtypes of patients would be automatically included in studies designed to test a priori hypotheses. In this case, “observations” would have a prominent “confirmatory” (or “refuting”) role.

2. Integration of experiments into routine clinical practice

Observational studies and pragmatic clinical trials conducted in the conditions of usual clinical practice are the main methods to conduct comparative effectiveness research and to generate real world evidence. Observational studies are usually conducted using databases and registries of patients. Pragmatic randomized trials include heterogeneous populations of patients and assess long term effectiveness of interventions in real life, but the required infrastructure and oversight generates extraordinarily high costs.

Today, one of the main challenges in clinical research is how to integrate experiments into routine clinical practice. The idea of conducting “*Randomized Database Studies*”, that was proposed fifteen years ago as a possible solution to combine the main strengths of randomized controlled trials (i.e., initial randomization) and registries (i.e., naturalistic follow-up) [53], may represent a new and disruptive paradigm in clinical research [54]. There are several recent examples of pragmatic trials that exploit routinely collected data to quickly demonstrate effectiveness in real-world care delivery systems [55, 56].

Electronic health records could also be used to conduct experiments in individual patients. N-of-1 studies are crossover trials, where the patient is his/her own control. Single patient trials are the only current vehicle for resolving clinical uncertainty about whether new and existing treatments are truly effective for a particular patient [57].

## Summary

**Figure 2:**
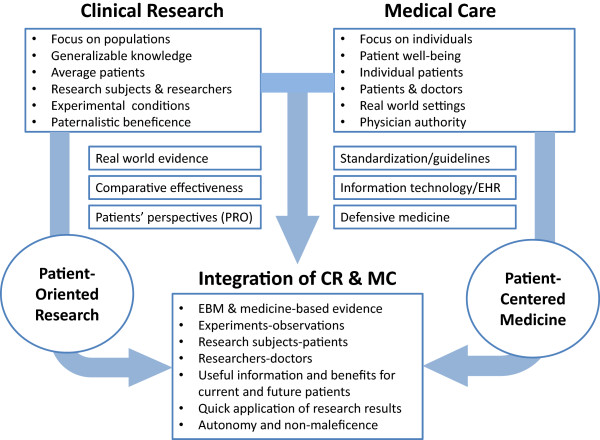
**Contributions of patient-oriented research and patient-centered medicine to the integration of clinical research and medical care.** Abbreviations: CR = clinical research; MC = medical care; PRO = patient-reported outcomes; EHR = electronic health records.

The substantial changes that are occurring within health care systems are contributing to blur the distinction between research and care. On one hand, clinical research is evolving towards the assessment of effectiveness under real life conditions, taking into account the patients’ perspectives and preferences. On the other hand, the standardization of clinical practice, the development of the new information technologies –mainly electronic health records -, and the practice of a defensive medicine, is accelerating the incorporation of typical elements of research into clinical practice. The development of *Patient Centered Medicine* and patient-oriented research represent a unique opportunity to integrate clinical research and medical care (Figure 2), but such integration appeals for the confluence of EBM and Patient Centered Medicine worlds, the rapprochement of experiments and observations, and the development of an integrated learning health system that contribute to generate knowledge and to produce benefits for individual and for average patients, today and in the future.

In order to achieve the integration, several ethical and methodological challenges should be met. Ethically, clinical research and medical care should evolve from a situation of paternalistic beneficence to a situation predominated by the principles of non-maleficence and patient autonomy. “*Patient-oriented informed consent* ”,“ *patient equipoise”* and *“altruism-based research”* are some of the potential solutions to practice a true patient-oriented research integrated into clinical practice. The application of *risk-based ethical oversight*, in which the level of ethical oversight is adapted to the risk for patients will be essential to integrate research and treatment.

Methodologically, the distance between clinical research and medical care should be attenuated in the same way as the distance between observations and experiments. Clinical research should yield results applicable to individual patients and medical practice should enable the possibility to learn from daily practice. Clinical research should evolve towards a *patient-oriented research*, a type of research that needs to rediscover the crucial role of careful observations and its confirmatory value. In a complementary way, experiments should be systematically integrated into daily medical practice. The implementation of *Randomized Database Studies* and single patient trials through electronic health records and decision-aids tools may contribute to decrease the cost of comparative effectiveness research, to accelerate the generation of new knowledge and, most importantly, to the fast translation of clinical research findings to present and future patients.

The successful integration of clinical research and medical care will require important cultural and organizational challenges. The alignment of incentives, goals, and metrics focused on improving patient-centered outcomes; the evolution towards patient-centered medical education; and the generation of evidence about the health benefits that integrated learning health care systems produce, are crucial elements to facilitate the transformation. But the key cultural challenge is that researchers, clinicians, health care managers, and patients to reevaluate the way they understand research and medicine.
